# DEMENTIA-FRIENDLY ENVIRONMENTS IN THE COMMUNITY – AN INTERNATIONAL PERSPECTIVE ON CONCEPTS AND INTERVENTIONS

**DOI:** 10.1093/geroni/igad104.0766

**Published:** 2023-12-21

**Authors:** Karin Wolf-Ostermann, Hilde Verbeek, Marie Boltz

## Abstract

Dementia is one of the major age-related societal challenges and causes enormous demands for persons living with dementia (PlwD). Although dementia is one of the major reasons for relocation into a nursing home, most PlwD want to live as long as possible in a familiar domesticity. Therefore, innovative care concepts for community-dwelling PlwD are being developed worldwide as alternative to traditional long-term care settings. Efforts are made to learn more about dementia-friendly environments in order to improve quality of care and quality of life of PlwD. This international symposium will therefore provide four presentations as well as a concluding discussion addressing new concepts for innovative care environments and measuring dementia-friendliness of the built environment as well as nonpharmacological interventions to support high quality person-centred care. The first presentation explores the functions of 24-hour green care farms as alternative to nursing homes and how these can be transferred to other care settings. The second presentation discusses the impact of moving from traditional care environments to innovative care settings for residents and staff. The third presentation presents results of a cluster randomized controlled trial in German shared housing arrangements where a complex nonpharmacological intervention led to significantly less hospital admissions for PlwD. And the final presentation describes an innovative approach to evaluate the wayfinding infrastructure in urban areas for PlwD and to promote dementia-friendliness in urban living structures. The discussant will synthesize the research findings and lead a discussion of research implications and future directions for policy and practice.

